# Combination of convalescent plasma therapy and repurposed drugs to treat severe COVID‐19 patient with multimorbidity

**DOI:** 10.1002/ccr3.3964

**Published:** 2021-02-22

**Authors:** Santa Kumar Das, Kamal Ranabhat, Suraj Bhattarai, Khem Bahadur Karki, Pradip Gyanwali, Hem Raj Paneru, Bipin Nepal, Shyam Prakash Dumre, Subhash Prasad Acharya

**Affiliations:** ^1^ Institute of Medicine Tribhuvan University Teaching Hospital Kathmandu Nepal; ^2^ Ministry of Health and Population Kathmandu Nepal; ^3^ Global Institute for Interdisciplinary Studies Kathmandu Nepal; ^4^ Grande International Hospital Kathmandu Nepal; ^5^ Central Department of Microbiology Tribhuvan University Kathmandu Nepal; ^6^ Institute of Tropical Medicine Nagasaki University Nagasaki Japan

**Keywords:** convalescent plasma therapy, COVID‐19 infection, multimorbidity, Nepal

## Abstract

Combination of convalescent plasma therapy and repurposed drugs such as dexamethasone and remdesivir could be beneficial for severe COVID‐19 patients with obesity and chronic diseases such as diabetes and hypertension.

## Graphical abstract

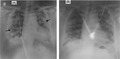

## 1 INTRODUCTION

COVID‐19 is a viral disease caused by severe acute respiratory syndrome coronavirus 2 (SARS‐CoV‐2), and its incubation period typically ranges from 2 to 14 days (98% of patients), with an average of 5 days.^1^ Although the majority of patients are either asymptomatic or with mild disease, in patients with chronic health conditions, symptoms may progress into severe pneumonia with almost 20% requiring hospitalization.^2^ Large scale studies that enrolled thousands of patients globally have reported comorbidities, particularly cerebrovascular disease, chronic obstructive pulmonary disease, chronic kidney disease, cardiovascular disease, hypertension, diabetes, cancer, and dyslipidemia as potential prognostic factors of severity and mortality in patients with COVID‐19 disease.^2^, ^3^

The epidemiology of SARS‐CoV‐2 infection is rapidly changing across the world. As of December 10, 2020, more than 68 million people are infected with SARS‐CoV‐2 infection worldwide, with over 1.5 million deaths.^4^ Standard treatment of COVID‐19 has not been established yet, although drugs such as dexamethasone and remdesivir have been repurposed for the treatment of moderate to severe COVID‐19 patients with some reported benefits.^5^, ^6^ Convalescent plasma has also been considered as a potential promising treatment modality for hospitalized COVID‐19 patients with a number of potential benefits: improved survival, improvement in symptoms, decreased risk of intubation for mechanical ventilation, decrease risk of intensive care unit (ICU) admission, shortened hospitalization time, and rapid reduction in viral load.^7^, ^8^ Transfusion of fresh‐frozen plasma from COVID‐19 convalescent patient (donor) allows transfer of neutralizing antibodies directed against SARS‐CoV2 antigens to the recipient, generating a passive immunization. However, the efficacy of convalescent plasma may not be better than other treatments, for example, remdesivir, in certain clinical endpoints. In a multicentric study conducted in Poland, the addition of remdesivir to convalescent plasma did not improve the effectiveness either.^9^

Like many countries worldwide, Nepal also faced rapid surge of COVID‐19 infection since early May 2020. As of 10 December, 245,650 people have been infected with 1,663 reported deaths and 1.73% of total cases requiring critical care.^10^ However, there are no enough data published in the literature from Nepal on COVID‐19 cases, their epidemiological dynamics, and treatment landscapes. Here, we present the first case of severe COVID‐19 infection who received convalescent plasma therapy, in addition to dexamethasone and remdesivir, and got fully recovered.

## 2 CASE DETAILS

A 60 years old male infected with SARS‐CoV‐2 virus, confirmed by reverse transcriptase‐polymerase chain reaction (RT‐PCR) test, was referred to our center on July 25, 2020, with the diagnosis of severe COVID‐19 disease with moderate acute respiratory distress syndrome (ARDS) for which he was hospitalized elsewhere for six days. He had a history of nonproductive cough and fever (recorded up to 101°F) for six days. He developed shortness of breath and worsening cough two days before presentation to Tribhuvan University Teaching Hospital, Kathmandu (TUTH). On physical examination upon arrival, he was found dyspneic with respiratory rate of 28/min, SpO2 of 93% with 40% O2 via venturi mask. On admission, his body temperature was 98°F, with pulse rate 90/minute, and blood pressure 150/90 mmHg (MAP = 109 mmHg).

The patient was obese (body mass index 35 Kg/m^2^) with multiple comorbidities: systemic hypertension for which he was taking Amlodipine + Losartan (2.5 mg + 50 mg), type 2 DM for which he was taking Metformin, and psoriasis for which he was on and off on steroids and recently took Methotrexate (last dose taken two weeks before presentation).

**FIGURE 1:**
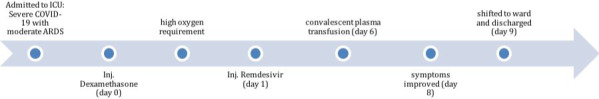
Timeline of patient management

On the day of admission (day 0), the patient was kept on high‐flow nasal cannula (HFNC) with oxygen flow maintained at 60 Liters/min and FiO2 titrated to target SpO2 >88%. That day, his oxygen requirement via HFNC increased from 40% to 60%. He was on restrictive fluid intake (targeting a negative fluid balance) and advised to lie down in prone position as much as possible. His laboratory parameters were all normal except decreased lymphocytes and elevated levels of liver alanine transaminase (ALT), serum urea, and urine albumin (Table 1). His medications included the following: injection Dexamethasone 6 mg q24h intravenous (IV), tablet Aspirin, Inj. Enoxaparin 80 mg SC q24h, Paracetamol for fever, and antihypertensive drugs continued. Metformin was stopped and switched to a regular insulin sliding scale. Despite these medications, he was restless with increased shortness of breath, tachypnoea (RR up to 28/minute), and increasing oxygen requirement. On day 1, the patient was given Inj. Remdesivir 200 mg stat followed by Inj. 100 mg q24h (Figure 1). His total leukocyte count increased on day 2 onward, along with increased C‐reactive protein (CRP), serum ferritin, and ALT levels (Table 1).

**TABLE 1:** Laboratory parameters of the patient (abnormal values in bold)

Parameters	Reference range (TUTH)	Day 0 (July 25, 2020)	Day 2	Day 3	Day 5	Day 6 (CPT)	Day 7
TLC, DLC (per mm^3^)	4000‐1100/ N55%‐75%, L20%‐40%, M2%‐8%	9600; N75 **L15**	**14 600**; N79 **L15**	**15 400**; N73 **L22**	**11 500**; N73 L20 M7	**12 500**; **N83** **L12**	
Hb (gm/dL)	12‐17	11.7	11.5	12.5	11.4	12	
Platelets (per mm^3^)	150000‐450000	250 000	273 000	286 000	322 000	353 000	
Urea (mmol/L)	1.5‐7.0	**8.3**	5.1	5.7	**8.3**	**7.3**	
Creatinine (µmol/ L)	60‐110	89	86	73	79	68	
Bilirubin total/ direct (µmol/L)	3‐21/0‐5	6/ 3	11/4	13/4	8/3	11/4	
ALT (U/L)	5‐45	**64**	**99**	**96**	42	52	
INR	≤1.1	**1.26**	**1.26**		**1.35**	**1.44**	
Urine albumin		**positive**					
Serum LDH (U/L)	<480	163			286		
Serum ferritin (ng/mL)	20‐250		**283**		**354**	**377**	**361**
CRP (mg/L)	<10		**90.0**		**50.9**	**37.9**	**23.2**
Procalcitonin (ng/mL)	<0.10		0.05		**0.18**	0.05	
D‐dimer (mg/L)	<0.50				0.28	**0.60**	0.37
FDP					negative		negative
IL‐6 Level (pg/mL)	≤4.40	4.40 (day 4 of admission)					
HIV 1/2; HCV; HBsAg		Nonreactive					
Blood culture (sample sent on day 3, reported on day 6)		no growth of pathogens					
Sputum gram stain and culture (sample sent on day 3, reported on day 6)		pus cells; epithelial cells (<10/LPF); *budding yeast cells* with pseudo‐hyphae (>25/LPF) suggestive of fungal infection. (Antifungal, tablet Voriconazole, was added to his medication list on day 6.)					

Abbreviations: ALT, alanine transaminase; CRP, C‐reactive protein; FDP, fibrinogen D protein; Hb, hemoglobin; HbsAg, hepatitis B surface antigen; HCV, hepatitis C virus; HIV, human immunodeficiency virus; IL, interleukin; INR, international normalized rate; LDH, lactate dehydrogenase; LPF, low‐power field; PT, prothrombin time; TLC, total leukocyte count; DLC, differential leukocyte count.

**FIGURE 2:**
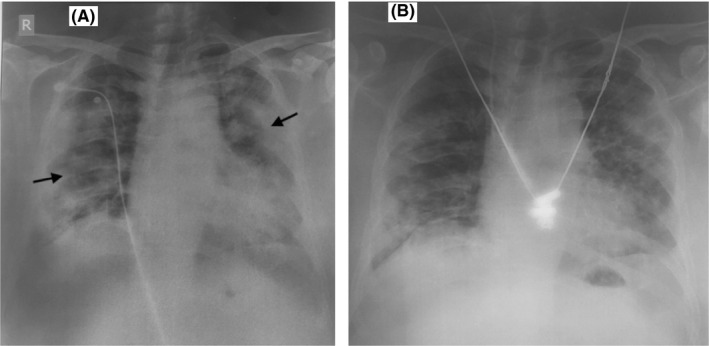
A, Chest X‐ray of the patient (anterior‐posterior view) showing bilateral lung infiltrates. B, Chest X‐ray of the patient (anterior‐posterior view) showing gradual improvement

Despite the first doses of two repurposed drugs, his oxygen requirement kept increasing up to 90% via HFNC. On day 3, Inj. Aztreonam was added empirically for suspected secondary bacterial pneumonia. It was selected among several other available antibiotics for its safety profile. In the background of worsening hypoxemia, increasing bilateral infiltrates on chest X‐ray, and elevated total leucocyte counts, there was a strong suspicion of secondary bacterial infection, particularly staphylococcal infection which is one of the most common lower respiratory tract gram‐positive bacterial infections reported among aging population in our center (Figure 2A, Table 1).^11^ Accordingly, Inj. Teicoplanin was added empirically while awaiting blood culture report (which came out negative) and sputum gram stain and culture results (later reported to have pus cells and fungal growth).

Although the patient was gradually feeling better on day 4 with decreased cough, decreased shortness of breath, and normal respiratory rate, he still had high oxygen requirement, with bilateral chest X‐ray infiltrates, and elevated inflammatory biomarkers reported on day 5 (CRP, serum ferritin, procalcitonin, D‐dimer) (Figure 2A, Table 1). The treating physicians then decided to transfuse plasma to the patient. Convalescent plasma was obtained from a blood group matched (B+) healthy donor—who recovered from SARS‐CoV‐2 infection, was asymptomatic for 26 days, had negative RT‐PCR report on July 04 (26 days before plasma collection), and gained high titer (IgG S/Co 421; neutralization antibody titer >1:250) estimated by Chemiluminescence Immunoassay Analyzer (CLIA) method in Vitros5600, Ortho clinical diagnostic machine. 200 mL of donor's plasma was successfully administered to the patient over 2 hours on day 6 of admission (July 31, 2020), that is, 12 days after onset of the patient's symptoms. No adverse effect of the intervention was reported.

The patient's oxygen requirement decreased gradually after convalescent plasma transfusion. His laboratory parameters improved on day 7: normal D‐dimer, decreased levels of CRP, serum ferritin, and procalcitonin (Table 1). On day 8, his oxygen saturation level was above 88% at 4 L/minute oxygen via nasal prongs, so his HFNC was discontinued. His chest X‐ray gradually improved (Figure 2B). The patient was shifted from critical care to the general ward on day 9 of his admission, that is, after 15 days of symptom onset, on oxygen at 3 L/min via nasal prongs. On the same day, the patient was transferred to another hospital on his personal wish for rehabilitation measures as part of ongoing treatment. During virtual and in‐person follow‐ups with the treating physicians in the following days, the patient was reported fully recovered.

## 3 DISCUSSION

Multimorbidity is a risk factor for SARS‐CoV‐2 infection and progression of COVID‐19 disease.^12^ Our patient was obese and had systemic hypertension, diabetes, and psoriasis. For these comorbidities, the patient was on regular medications including Methotrexate (an immunosuppressant) for psoriasis. In light of reported benefits of repurposing existing drugs and old treatments for treating COVID‐19 infection, the patient received dexamethasone on day 0 and remdesivir on day 1 of admission.^5^, ^6^ Additionally, the patient received convalescent plasma on day 6 of admission and showed signs of clinical improvement after two days of transfusion. This was the first ever convalescent plasma transfusion for SARS‐CoV‐2‐infected patients in Nepal which became successful.^13^

Convalescent plasma isolated from COVID‐19‐recovered individuals is a good source of neutralizing antibody which provides passive immunity.^7^ If administered to severe COVID‐19 patients, the donor's plasma with sufficient neutralizing antibody levels may reduce viral load and lower the mortality risk.^14^ A case series of five critically ill COVID‐19 patients with ARDS in China showed that administration of convalescent plasma containing high titers of neutralizing antibody led to clinical improvement of all patients with ARDS resolved, increased neutralizing antibody titers, decreased viral load, and early discharge.^15^

Our patient received convalescent plasma after 12 days of his onset of illness. A study reported that convalescent plasma administered before 14 days of disease onset demonstrates a remarkable improvement in patient condition compared to that administered after 14 days.^14^ Administration of convalescent plasma early on in the disease course is expected to increase lymphocyte count and decrease CRP levels, with remarkable resolution of the pulmonary lesions on imaging. The same study reported that convalescent plasma combined with other treatments, such as antiviral therapy and supportive care, improved clinical outcomes in COVID‐19 patients.^14^ Our patient was on Methotrexate (immune‐suppressant) for his psoriasis, with the last dose taken two weeks before hospitalization. A multi‐center study in Italy found that the incidence rate of death due to COVID‐19 disease was lower in patients with psoriasis (under biological therapy) than in general population, suggesting no need of discontinuing ongoing therapy.^16^ Additionally, during hospitalization, the patient was treated with higher than normal dose of prophylactic anticoagulant (Enoxaparin 80mg q24h).^17^ It was unknown whether convalescent plasma would interact with such biological and medical treatments in COVID‐19 patients with underlying chronic diseases.

Our patient had a significant clinical improvement after two days of transfusing convalescent plasma. A randomized clinical trial reported higher levels of SARS‐CoV‐2 antibody titers in convalescent plasma group as compared to placebo group after two days of intervention.^18^

Convalescent plasma therapy alone may not be the effective treatment modality for reducing disease severity, mortality risk, and hospital stay in patients with COVID‐19 infection.^19^, ^20^ Nontargeted antibody transfusion has its own risks such as antibody‐dependent enhancement of infection (ADE) and immune modification leading to reinfection, risks associated with transfusion such as transmission of blood borne infections (eg, HIV, HBV, and HCV), allergic reaction, transfusion associated lung injury (TRALI) and cardiac overload, and ABO incompatibility.^21^ Despite these risks, passive antibody administration could be the only method of rendering immediate immunity in highly immunocompromised and multimorbid patients who would rather fail to achieve an adequate immune response following active immunization or vaccination.^21^

Considering the conflicting results of multiple convalescent plasma clinical trials and the recent findings of the WHO Solidarity trial that there is no benefit of repurposing antiviral drugs (remdesivir, hydroxychloroquine, lopinavir, and interferon regimens) in hospitalized patients with COVID‐19, the combination therapy of convalescent plasma and repurposed drugs could be an alternative solution.^19^, ^20^, ^22^ Large scale studies are required to determine whether the combination therapy would lead to immediate improvement and long‐term immunity in multimorbid and susceptible patients.

## 4 CONCLUSION

The combination therapy of convalescent plasma and repurposed drugs may be helpful in reducing the complications of COVID‐19 disease in patients with multimorbidity and immunocompromised status. With the rising number of SARS‐CoV‐2 recovered patients who are presumably a good source of neutralizing antibodies for immunity, convalescent plasma could be a cost‐effective, safe, and easily accessible treatment for severe COVID‐19 patients in low‐resource settings.

## CONFLICT OF INTEREST

None.

## AUTHOR CONTRIBUTIONS

SKD, KR, and SPA: conceptualized the case study. SKD, KR, SB, and HRP: wrote the first manuscript draft and reviewed the literature. KBK, PG, BN, and SPD: provided critical feedback to the draft. All authors read and approved the final version of the manuscript.

## ETHICAL APPROVAL

Written informed consent was obtained from the patient for publishing his case details along with his chest images. The first author, also the treating physician, is responsible and guarantor of the patient consent.

## Data Availability

The data that support the findings of this study are available from authors upon reasonable request.
